# Quantifying entropic stabilization of the structurally tolerant ionic phase of lithium alanate

**DOI:** 10.1038/s42005-026-02726-0

**Published:** 2026-06-18

**Authors:** Omer Tayfuroglu, Marco Krummenacher, Jonas A. Finkler, Hannes Huber, Stefan Goedecker

**Affiliations:** 1 Department of Physics, University of Basel, Basel, Switzerland; 2 Department of Chemical and Biological Engineering, Koc University, Istanbul, Turkey; 3 Department of Chemistry and Bioscience, Aalborg University, Aalborg, Denmark

**Keywords:** Theory and computation, Structural materials

## Abstract

In materials simulations, formation energies can provide valuable information about the stability of different crystal polymorphs. However, at finite temperatures, it is often essential to go beyond simple energetics and include free energies in the analysis, as many crystal phases are stabilized by entropic contributions. Therefore, solid-solid phase transitions at finite temperatures are common. Traditionally, free energies of crystalline solids are computed through an analysis of the phonon band structure, which is calculated within the harmonic approximation considering only the crystalline unit cell and its periodic replications. Configurational contributions to the free energy are typically only taken into account for systems such as high-entropy alloys, where the multiplicity can be calculated analytically. We present a numerical approach for calculating configurational entropy that is applicable to essentially any system. By a combination of machine-learned interatomic potentials and efficient structure search methods, we are able to obtain a huge number of inherent structures and study their influence on the free energy of the system. To obtain these structures, it is necessary to generate large periodic cells that can represent not only the perfect crystalline structure but also a very large number of defective structures. Reliable estimates of phase transition temperatures can only be obtained if the configurational density of states is well converged with respect to this cell size. We illustrate this effect for lithium alanate, where the configurational entropy contribution of the structurally tolerant ionic phase reduces the transition temperature to the polymeric phase by 150 K.

## Abstract (web summary)

With a few known exceptions such as high entropy alloys, solids are generally considered to reside in one minimum of the potential energy surface. In this study of lithium alanate the authors show that there is a defect tolerant phase in which the material is residing in a very large number of minima, leading to a strong entropic stabilization of this phase.

## Introduction

Modern structure prediction methods^1–18^ are able to find many metastable structures for solids, clusters, and other condensed matter systems. The most reliable energetic ordering of metastable structures is generally obtained from density functional theory calculations. Although energetic ordering can depend on the exchange-correlation functional, different high-quality functionals agree with each other in most cases, as well as with results obtained from beyond-DFT methods^19,20^. Despite this agreement, the experimentally observed stable phase is still frequently different from the theoretical zero temperature ground state.

Actually, finite temperature effects are frequently non-negligible and the ordering according to the finite temperature free energy can be different from the ordering of the zero temperature energy. To take finite-temperature effects into account, the vibrational free energy is typically calculated for low-energy structures to obtain the energetic ordering at the temperature of interest.

**Fig. 1:**
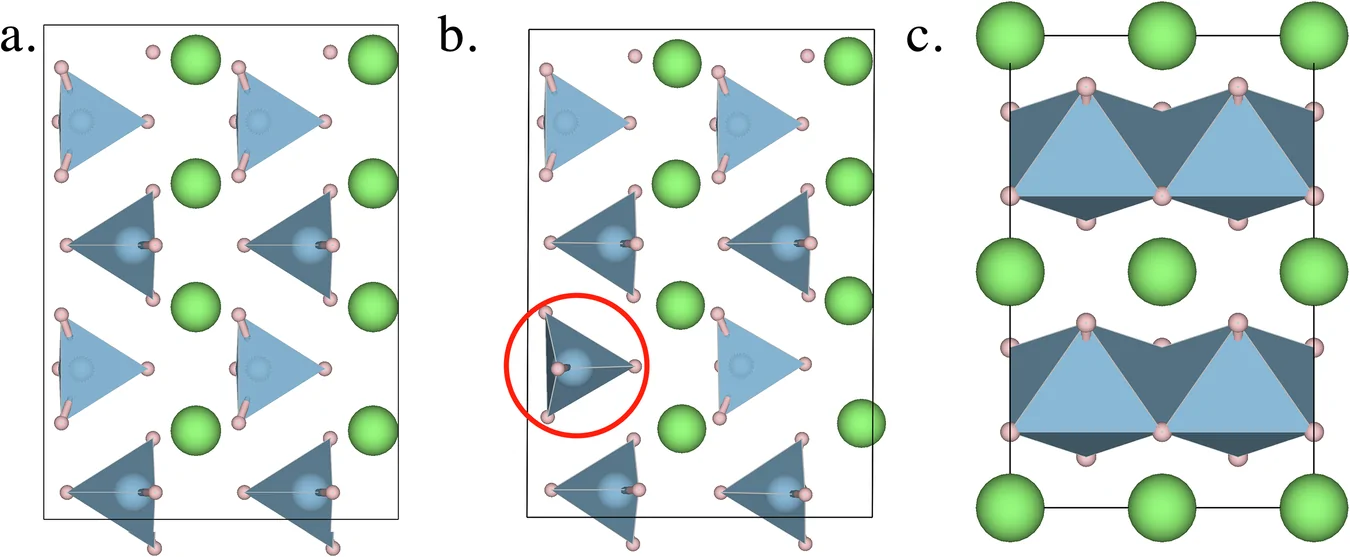
Different structures of lithium alanate. **a**. The lowest-energy ionic structure, **b** Ionic structure obtained from the lowest ionic structure in (**a**) by rotating the encircled *AlH*_4_ tetrahedron, **c** lowest-energy polymeric polymorph. Rotating the tetrahedron raises the energy only by 33 meV per formula unit of LiAlH_4_ in the 48-atom unit cell. The images were generated using the VESTA software^60^.

The standard way to calculate the vibrational free energy is in the harmonic or quasi-harmonic approximation^21^. At high temperatures, the system can visit regions of its catchment basin that are not any more accurately described by pure quadratic terms. Such anharmonic effects can also be taken into account^22–24^, albeit at high computational cost. Independent of whether and how such anharmonic effects are taken into account, most methods to calculate free energies assume that the system remains within a single catchment basin. However, to take configurational entropy into account, several catchment basins must be included in the calculation of the free energy. The influence of a few structures on thermodynamic properties has been studied^25,26^. Systems that are dynamically unstable in the small primitive cell relax into two or more dynamically stable structures in larger cells. Configurational entropy based on a large number of structures has recently been identified as a driving force for the formation of two classes of materials, high-entropy alloys^27,28^ and high-entropy oxides^29^. The theoretical description of the entropy of both materials is based on a simple lattice model where one calculates by basic combinatorics in how many ways different elements can be distributed on the lattice sites. If there are more than four or five different elements, there is a huge number of possible distributions, i.e., a very high multiplicity and the entropy *S*, which is obtained from this multiplicity *W* by the famous Boltzmann formula $S\,=\,{k}_{{{\rm{B}}}}ln(W)$, dominates the physical properties of the material. The effects of including vibrational entropy contributions in such lattice models have also been studied^30^. For ordinary materials entropy differences between different phases are generally assumed to be on the order of 0.2 *k*_*B*_^30^, while for high-entropy alloys they can rise up to two *k*_*B*_. In this article, we develop a method that can calculate configurational entropies for arbitrary systems and use it to show that configurational entropy is also an important driving force in a wider class of solids for which lattice models are not applicable. We consider lithium alanate (LiAlH_4_), which has been studied extensively because of its promising properties as a hydrogen storage material. The entropy difference between its two phases is even higher than for the high-entropy alloys, namely 4 *k*_*B*_. The experimentally found phase at ambient conditions has ionic character^31^. As shown in Fig. 1, negatively charged ${{{\rm{AlH}}}}_{4}^{-}$ tetrahedra and positive lithium cations occupy spatially separated alternating sites that are reminiscent of textbook ionic structures. Depending on how the anions and cations are arranged, they are referred to as alpha, beta and gamma phases^32^. However, in a theoretical structure prediction by Amsler et al.^33^, a polymorph with space group P2_1_/c was identified as the lowest-energy structure. These polymeric structures consist of octahedra, where 6 hydrogen atoms occupy the corners and the aluminum is at the center. To satisfy the 1–4 stochiometry, the octahedra cannot be isolated but have to share corners or edges. As shown in Fig. 1, the octahedra therefore form polymeric structures. We have repeated the calculations of Amsler et al. with a highly accurate modern exchange correlation functional, SCAN^34,35^, which was not yet available at the time of the structure predictions by Amsler et al. However, also with this functional, the polymeric phases are the lowest in energy.

If one includes the zero-point energy together with the vibrational entropy, one also finds with all functionals that the ionic phases become the lowest in free energy upon increasing the temperature.

In the ionic phases, the tetrahedra can make low-frequency vibrational movements that explore a large phase space volume and thus increase the vibrational entropy. In the case of the polymeric phases, the links between the octahedra prevent such vibrations and hence give rise to a lower vibrational entropy. As shown later in this work, this stabilization of individual polymorphs of the ionic phase by the vibrational entropy gives rise to a phase transition at about 700 K between the lowest-energy polymeric and ionic polymorphs.

In the following, we show that an important contribution to the free energy is missed if only the vibrational entropy is considered while the configurational entropy is neglected.

Zhao et al.^36^ have recently introduced the notion of polymorphous structures. Polymorphous structures cannot be described by standard small unit cells. Many unit cells, which differ slightly from each other, are needed to construct supercells that can describe the geometric ground state. In this way, the short-range translational symmetry of a description with small unit cells is broken. In the case of the halide perovskites studied by Zhao et al., the driving force for the breaking of the short-range periodicity is purely energetic. In our case, the lowest-energy alanate structure can be described by a small unit cell, but many metastable structures are slightly higher in energy and can only be represented by large cells because their short-range translational symmetry is broken. At sufficiently high temperature, a phase consists of many such metastable structures. So we also end up with polymorphous structures, but the driving force is entropic instead of energetic.

In this work, we use the superposition method that was originally proposed by Stillinger^37,38^. This method was recently applied under the name of “zentropy method” to explain the apparent structural differences obtained by x-ray diffraction and x-ray absorption^39^. In this case, 24 symmetry equivalent local minima were taken into account.

## Results

**Fig. 2:**
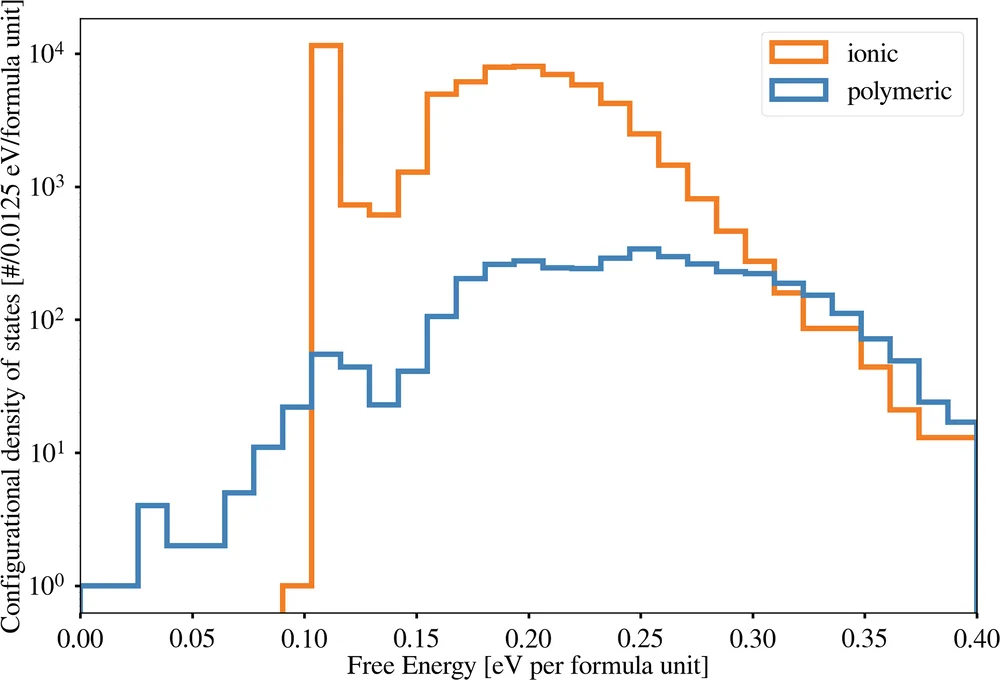
The density of states per formula unit of LiAlH_4_ as a function of the free vibrational energy at 0 K relative to the ground state, subdivided into the contributions from the two phases. A structure was supposed to be ionic if its average aluminum coordination number was less than five and polymeric otherwise.

The ionic phase of lithium alanate is a structurally tolerant phase since the isolated tetrahedra can be rotated to find other low-energy structures (see Fig. 1). In addition, AlH_4_ anions and Li cations can adopt several prototype ionic crystalline structures such as rock salt, wurzite and caesium chloride, which are also close in energy.This leads to a very high density of states above about 0.1 eV per formula unit of LiAlH_4_ in Fig. 2.

**Fig. 3:**
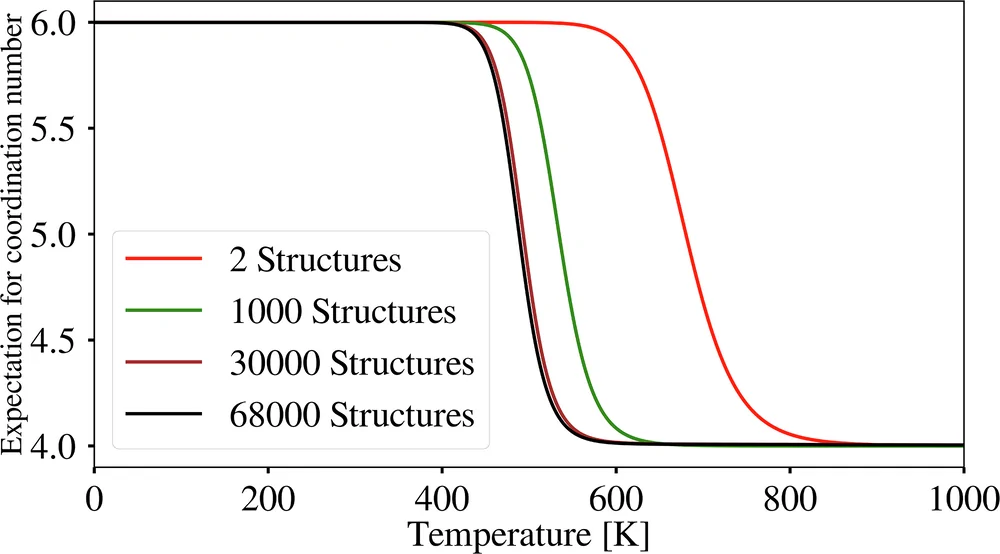
The expectation value of the coordination number taking into account different numbers of metastable structures. Convergence with respect to this number is reached when some 68'000 structures are included in Eq. (3). The red curve is the result of a conventional treatment where only the vibrational entropy of the 2 lowest structures of each phase is taken into account.

Energetically close polymorphs have also been found in structure predictions of simpler ionic materials such as lithium chloride^40^, where the cations and anions are single atoms and not polyhedra. In contrast, polymeric polymorphs have a much smaller number of structures in the region above 0.1 eV per formula unit of LiAlH_4_, but exhibit a non-vanishing density in the lower-energy region, where no ionic structures can be found (Fig. 2). The configurational density of states is obtained by performing Minima Hopping^4–7,41^ runs to find a huge number of metastable structures. A Minima Hopping run consists of alternating local geometry optimizations, which identify stable structures at the bottom of catchment basins, and short MD trajectories that allow the system to jump from one catchment basin to another. By limiting the kinetic energy and initiating the MD trajectories along low-curvature directions^6,7^, the jumps occur preferentially over low energy barriers and correspond to collective global atomic rearrangements. This behavior contrasts with ordinary MD, which tends to produce predominantly local atomic rearrangements. In a Minima Hopping run, one starts from an arbitrary configuration and in the first phase of the run, the energies go down in the majority of hops until the global minimum is found. Afterwards, in the second phase, Minima Hopping slowly leaps up in energy, locating higher and higher local minima. Once the majority of all physically reasonable local minima has been found, the feedback mechanism of Minima Hopping increases the kinetic energy of the MD escape trajectory for every revisited minimum, and the system finally explodes. The number of metastable structures found in such a second phase of Minima Hopping was plotted against the vibrational free energy of these metastable structures in Fig. 2. The decrease of the sampled density of states at higher energies arises because the Minima Hopping algorithm preferentially explores the physically relevant low-energy regions of the potential energy surface. About half of the minima within 0.1 eV/f.u. of the ground state are visited at least twice and the minima closest to the ground state are visited many times once more than 1000 inherent structures are sampled. In agreement with this indicator, the transition temperature starts to go down continuously with this number of inherent structures and reaches full convergence for 30,000 structures as shown in Fig. 3.

In order to be able to represent various low-energy structures, it is necessary to use cells that are much larger than the standard crystalline unit cells. However, the number of metastable structures is increasing strongly with respect to the number of atoms in the simulation cell. Therefore, the cell should not become too large. In the Supplementary Note 2, we show that well-converged thermodynamic quantities can be obtained once the free energy starts to increase linearly with the cell size. We found that a 48-atom cell is a good compromise in practice. While it is large enough that both ionic and polymeric phases can coexist, the local minima of the potential energy surface can still be sampled well enough to obtain converged results, as can be seen from Fig. 3. To obtain a reliable configurational density of states, some 68,000 distinct structures were found. The overlap matrix fingerprint^42^ was used to eliminate duplicate structures and to make sure that the 68,000 structures are really distinct. To further characterize the structural diversity of these configurations, we performed a statistical analysis of their structural similarity by computing overlap matrix fingerprint distances from the ground state structure. As shown in Supplementary Fig. 3, structural differences correlate on average well with energy differences and the polymeric and ionic phases occupy distinct regions of configurational space. Since such a large number of metastable configurations cannot be handled on the DFT level, we constructed a machine-learned potential energy surface for lithium alanate using the message passing NequIP^43^ potential. The reference data was generated with ab-initio MDs. Although we study lithium alanate only at zero pressure, we performed ab-initio MD simulations with pressures up to 6 GPa with VASP^44–48^. This, together with a temperature range of 100 to 2000 K, ensured that we have many configurations in our training data set where two atoms come rather close. Thus, the machine-learned potential can learn how to treat short interatomic distances, which stabilizes the Minima Hopping runs with our machine-learned potentials. Energy and force evaluations were performed using VASP with the r2SCAN meta-GGA^35^ exchange-correlation functional. More detailed information on the construction of the machine-learned potentials can be found in the “method” section.

Various partitioning schemes for configurational space have been proposed, such as those described in ref. ^49^. We use the approach of Stillinger^38^ where the configurational space is partitioned into catchment basins around the so-called inherent structures, which are simply the local minima.

A catchment basin of an inherent structure is defined as the region of the potential energy surface with the property that, if a point inside this region is used as a starting point for a steepest descent iteration with a small step size, the steepest descent leads to this inherent structure. The catchment basins defined in this way are typically very long in the direction of the soft modes and very short in the direction of the hard modes. The distance between the inherent structures can therefore be shorter than vibrational distances along the soft modes in the catchment basin. This, however, does not hinder a unique partitioning of the configurational space. We calculate the partition function *Z* by decomposing it into contributions from the catchment basins of the metastable structures. Since a metastable structure consists of several vibrational quantum states, we use in the partition function not the energies of the metastable structures, but the corresponding free energies, which include the vibrational contribution calculated within the harmonic approximation as implemented in the Atomic Simulation Environment^50^. This procedure is known as the quantum superposition method^51^ and has been successfully applied to many systems^38,52^.

1 $$Z(\beta )=\mathop{\sum }\limits_{i}\frac{1}{{g}_{i}}\exp (-\beta {F}_{i}(\beta ))\,.$$

*F*_*i*_(*β*) is the free energy of configuration *i* calculated from the total energy of the configuration and its vibrational frequencies in the standard way and *g*_*i*_ is the order of the point group. The sum is in principle over all metastable structures, but in practice only over the sampled structures. A thermodynamic expectation value *a* of a quantity *a*_*i*_ can then be calculated as

2 $$a=\frac{{\sum }_{i}{a}_{i}\frac{1}{{g}_{i}}\exp (-\beta {F}_{i}(\beta ))}{{\sum }_{i}\frac{1}{{g}_{i}}\exp (-\beta {F}_{i}(\beta ))}.$$

The probability of finding the system in phase *α* is then given by the expectation value of an integer-valued function *a*_*i*_ which is equal to one if the system is in this phase and zero otherwise. Since many structures contain a mixture of both types of polymorphs, assigning them to one phase or the other can be ambiguous. We therefore base our analysis instead on the expectation value of some collective variable as a function of temperature. The coordination number of the central aluminum atoms with respect to the surrounding hydrogen atoms turns out to be an excellent quantity for characterizing the different phases. It is four for the ionic phase, where the aluminum atom is at the center of a hydrogen tetrahedron, and six for the polymeric phase, where the aluminum atom is at the center of a hydrogen octahedron. For some 100 structures in the region above 0.13 eV per formula unit (see Fig. 2), an average coordination number of 5 was observed, which resulted from either mixed phases that are partly ionic and partly polymeric or from five-fold coordinated Al atoms, which were also observed in ref. ^53^.

The thermodynamic expectation value *n*(*β*) of the coordination number can be calculated as

3 $$n(\beta )=\frac{{\sum }_{i}{n}_{i}\frac{1}{{g}_{i}}\exp (-\beta {F}_{i}(\beta ))}{{\sum }_{i}\frac{1}{{g}_{i}}\exp (-\beta {F}_{i}(\beta ))}.$$

where *n*_*i*_ is the average coordination number of aluminum atoms in the structure *i*. This expectation value is plotted in Fig. 3 as a function of the temperature *T* = 1/(*k*_*b*_*β*) for several scenarios. If one includes only the lowest ionic and polymeric structures, the phase transition from polymeric to ionic occurs at about 680 K. This is what is typically done if the free energies, obtained from the total energies plus the vibrational free energies, are compared to determine which polymorph has the lower free energy. Including the lowest 19 structures lowers the transition temperature to 640 K. Including all the structures gives a further very significant lowering of the transition temperature to 490 K. Thus we can observe that it is essential to include a large number of structures to converge to the exact transition temperature. Hence, the probability of finding a given phase is not only related to the properties of the lowest-energy polymorphs in the two phases, but also depends on the nature of the defective structures in each phase. If there are many low-energy defective structures in one phase whose vibrational free energies differ by some *k*_*B*_*T*, i.e., if this structural motif is structurally tolerant, it will be stabilized thermodynamically. Since the density of states differs in this temperature range by nearly 2 orders of magnitude, the entropy gain of the ionic phase with respect to the polymeric phase is about 4*k*_*B*_.

In order to support the above analysis, we also performed MD simulations. In our variable cell shape MD simulations at 700 K with the NequIP potential, the 96-atom system undergoes, after some 250 ps, a phase transition from a polymeric phase to an ionic phase. Transitions in the opposite direction were never observed, even in longer MD runs. This demonstrates that the ionic phase has a lower free energy at this temperature and that the transition temperature must therefore be below 700 K. A more accurate determination of the transition temperature is not possible with MD simulations of affordable duration. Relaxing structures along the MD trajectories revealed a huge number of distinct local minima with ionic character, confirming the huge density of states of the ionic phase. MD runs at 500 K also revealed that transitions from one catchment basin into another one are rare events. The MD trajectory typically remains in one catchment basin for some 2 ps. During this time, it oscillates about 60 times back and forth within this catchment basin without escaping from it. In contrast to our superposition method, MD-based methods are, however not able to disentangle the vibrational and configurational contributions to the free energy. By revealing which contributions to the free energy are of importance, the superposition approach can lead to a deeper understanding of the system.

## Conclusion

We have shown that a thermodynamic stabilization of certain phases by configurational entropy occurs not only in high-entropy alloys and high-entropy oxides but also in lithium alanate. In this complex material, the number of possible structures cannot be obtained by simple combinatorics, but instead requires the numerical determination of a very large number of metastable structures. In lithium alanate, a structurally tolerant ionic phase competes with a structurally more rigid polymeric phase. The large configurational density of states of the structurally tolerant ionic phase stabilizes this phase and leads to a significant reduction of the transition temperature to the polymeric phase. The temperature interval within which the polymeric phase is the thermodynamic ground state thus becomes smaller, while the ionic phase becomes entropically stabilized.

The large density of states arises from the fact that the ionic building block, the lithium cations and the AlH_4_ anions, can be arranged in many different ways without increasing the energy too much. To theoretically establish entropic stabilization, it is necessary to generate a sufficiently large number of defective structures in periodic cells that are much larger than the crystalline cells used in standard calculations. This is not possible with standard DFT methods because of the high computational cost, but it has now become feasible with machine-learned potential energy surfaces. Since standard methods for calculating free energies do not take into account configurational entropy contributions, it is to be expected that this type of stabilization can be found in many more materials where one phase is structurally more tolerant than the other. This phase will then be entropically stabilized, potentially with a significant impact on the transition temperature. Our results also show that the quantum superposition method can give deep insight into the mechanisms of a coupled configurational and vibrational entropic stabilization. In this study, we have considered only the case of zero pressure. By substituting free energies by Helmholtz free energies in Eq. (1), the method can, however, be straightforwardly generalized to finite pressures.

## Methods

### Development of machine learned-potentials (MLPs)

For this work, two types of machine-learned potentials (MLPs), namely HDNNPs and NequIP, were developed using an iterative process. To train both, a data set was first generated by selecting uncorrelated configurations as initial reference structures from multiple ab initio molecular dynamics (AIMD) simulations for both ionic and polymeric LiAlH_4_. In order to enhance the exploration of the configurational space, AIMD simulations were performed on a temperature-pressure grid, combining temperatures of 100, 300, 500, 1000, 1500, and 2000 K with pressure conditions of 0, 1, 2, 4, and 6 GPa. In a next step the MLPs were tested by performing NPT-MDs with the LAMMPS software ^54,55^ at different temperatures and pressures. Prior to the NPT-MD simulations, NVT equilibration were performed for 100 ps at different temperatures of interest using The Nosé–Hoover thermostat. To allow for changes in the cell shape, we used variable cell shape molecular dynamics^56^. In this method, each dimension was controlled using its individual stress components as driving forces, with a specified scalar pressure applied as the external normal stress. A 0.5 fs time step was also used for the Nosé–Hoover thermostat and barostat simulations. The damping constants were set to 0.1 ps for both the thermostat and the barostat. The MD trajectories generated were then used to refine the initial training data set. For selecting structures to add to the data set two different criteria were applied (i) atom-centered symmetry functions (ACSF)^57,58^ to identify how far the configurational distance is to the initial training data set and (ii) the uncertainty of the energy to a second MLP trained on the same training data.

### DFT calculations

AIMD, relaxation and single-point DFT calculations were performed using the projector augmented wave (PAW) method as implemented in the Vienna Ab initio Simulation Package (VASP) ^44–48^. A plane-wave basis set with a cutoff energy of 800 eV was used within the PAW formalism. The regularized-restored strongly constrained and appropriately normed (r2SCAN) meta-GGA^35^ exchange-correlation functionals were used, with a reciprocal space sampled using *Γ*-centered k-grid meshes with spacings of 0.2 Å^−1^. All calculations were spin unpolarized and relaxations were performed until the energy and residual atomic forces converged to 10^−7^ eV and 10^−3^ eVÅ^−1^, respectively. The above described process was applied to construct two types of MLPs, a Behler type second generation high dimensional neural network potential (HDNNP)^57^, implemented using the n2p2 package^59^ and a message passing NequIP ^43^ potential.

### Structure sampling with MLP-based Minima Hopping calculations

Minima Hopping^4–7,41^ runs coupled to both HDNNPs and NequIP were performed to find some 68,000 distinct meta–stable structures for cells containing 48 atoms. Duplicate structures were eliminated from our data sets using overlap matrix based fingerprints^42^. For this purpose we used an overlap matrix constructed from s-orbitals together with a cutoff radius of 8 Bohr and a fingerprint threshold distance of 0.008. As discussed in Supplementary Note 1, because of its more accurate forces (Supplementary Figs. 1–2), the NequIP potential was then used to calculate the free vibrational energies of all the structures based on harmonic vibrational frequencies that were obtained by finite differences as implemented in the Atomic Simulation Environment (ASE)^50^.

## Supplementary information

Supplementary information

## Data Availability

The data including the 68,000 minima from the Minima Hopping runs on the NequiP potential as well as the machine learning dataset are available on GitHub: https://github.com/KrumaKruma/Li-Al-H4.git.

## Code availability

The Minima Hopping code is publicly available on GitHub: https://github.com/KrumaKruma/MinimaHopping.git. The replica exchange code is available from the corresponding author upon request.

## References

[1] Kirkpatrick, S., Gelatt, C. D. & Vecchi, M. P. Optimization by simulated annealing. *Science* **220**, 671–680 (1983). 10.1126/science.220.4598.671 17813860

[2] Wales, D. J. & Doye, J. P. K. Global optimization by basin-hopping and the lowest energy structures of Lennard-Jones clusters containing up to 110 atoms. *J. Phys. Chem. A* **101**, 5111–5116 (1997).

[3] Doye, J. P. K. & Wales, D. J. Thermodynamics of global optimization. *Phys. Rev. Lett.* **80**, 1357–1360 (1998).

[4] Goedecker, S. Minima hopping: an efficient search method for the global minimum of the potential energy surface of complex molecular systems. *J. Chem. Phys.* **120**, 9911–9917 (2004). 10.1063/1.1724816 15268009

[5] Amsler, M. & Goedecker, S. Crystal structure prediction using the minima hopping method. *J. Chem. Phys.* **133**, 224104 (2010). 10.1063/1.3512900 21171680

[6] Sicher, M., Mohr, S. & Goedecker, S. Efficient moves for global geometry optimization methods and their application to binary systems. *J. Chem. Phys.* **134**, 044106 (2011). 10.1063/1.3530590 21280686

[7] Roy, S., Goedecker, S. & Hellmann, V. Bell-Evans-Polanyi principle for molecular dynamics trajectories and its implications for global optimization. *Phys. Rev. E* **77**, 056707 (2008). 10.1103/PhysRevE.77.056707 18643195

[8] Pickard, C. J. & Needs, R. J. High-pressure phases of silane. *Phys. Rev. Lett.* **97**, 045504 (2006). 10.1103/PhysRevLett.97.045504 16907590

[9] Martoňák, R., Laio, A. & Parrinello, M. Predicting crystal structures: the Parrinello-Rahman method revisited. *Phys. Rev. Lett.* **90**, 075503 (2003). 10.1103/PhysRevLett.90.075503 12633242

[10] Johnston, R. L. Evolving better nanoparticles: genetic algorithms for optimising cluster geometries. *Dalton Trans*. **22**, 4193–4207 (2003).

[11] Oganov, A. R. & Glass, C. W. Crystal structure prediction using ab initio evolutionary techniques: principles and applications. *J. Chem. Phys.* **124**, 244704 (2006). 10.1063/1.2210932 16821993

[12] Bhattacharya, S., Levchenko, S. V., Ghiringhelli, L. M. & Scheffler, M. Stability and metastability of clusters in a reactive atmosphere: theoretical evidence for unexpected stoichiometries of Mg_*M*_O_*x*_. *Phys. Rev. Lett.* **111**, 135501 (2013). 10.1103/PhysRevLett.111.135501 24116790

[13] Vilhelmsen, L. B. & Hammer, B. A genetic algorithm for first principles global structure optimization of supported nano structures. *J. Chem. Phys.* **141**, 044711 (2014). 10.1063/1.4886337 25084941

[14] Zhu, Q., Oganov, A. R., Lyakhov, A. O. & Yu, X. Generalized evolutionary metadynamics for sampling the energy landscapes and its applications. *Phys. Rev. B* **92**, 024106 (2015).

[15] Curtis, F. et al. Gator: A first-principles genetic algorithm for molecular crystal structure prediction. *J. Chem. Theory Comput.* **14**, 2246–2264 (2018). 10.1021/acs.jctc.7b01152 29481740

[16] Glass, C. W., Oganov, A. R. & Hansen, N. USPEX—evolutionary crystal structure prediction. *Comput. Phys. Commun.* **175**, 713–720 (2006).

[17] Wang, Y., Lv, J., Zhu, L. & Ma, Y. CALYPSO: a method for crystal structure prediction. *Comput. Phys. Commun.* **183**, 2063–2070 (2012).

[18] Falls, Z., Avery, P., Wang, X., Hilleke, K. P. & Zurek, E. The XtalOpt evolutionary algorithm for crystal structure prediction. *J. Phys. Chem. C.* **125**, 1601–1620 (2021).

[19] Peverati, R. & Truhlar, D. G. Exchange-correlation functional with good accuracy for both structural and energetic properties while depending only on the density and its gradient. *J. Chem. Theory Comput.* **8**, 2310–2319 (2012). 10.1021/ct3002656 26588964

[20] Finkler, J. A. & Goedecker, S. Experimental absence of the non-perovskite ground state phases of mapbi 3 explained by a funnel hopping Monte Carlo study based on a neural network potential. *Mater. Adv.* **4**, 184–194 (2023). 10.1039/d2ma00958g PMC9812259 36685989

[21] Fultz, B. Vibrational thermodynamics of materials. *Prog. Mater. Sci.* **55**, 247 – 352 (2010).

[22] Hooton, D. LI. a new treatment of anharmonicity in lattice thermodynamics: I. *Lond. Edinb. Philos. Mag. J. Sci.* **46**, 422–432 (1955).

[23] Frenkel, D. & Ladd, A. J. C. New Monte Carlo method to compute the free energy of arbitrary solids. application to the FCC and HCP phases of hard spheres. *J. Chem. Phys.* **81**, 3188–3193 (1984).

[24] Monacelli, L. et al. The stochastic self-consistent harmonic approximation: calculating vibrational properties of materials with full quantum and anharmonic effects. *J. Condens. Matter Phys.* **33**, 363001 (2021). 10.1088/1361-648X/ac066b 34049302

[25] Kadkhodaei, S., Hong, Q.-J. & van de Walle, A. Free energy calculation of mechanically unstable but dynamically stabilized bcc titanium. *Phys. Rev. B* **95**, 064101 (2017).

[26] Thomas, J. C. & Van der Ven, A. Finite-temperature properties of strongly anharmonic and mechanically unstable crystal phases from first principles. *Phys. Rev. B* **88**, 214111 (2013).

[27] Cantor, B. Multicomponent high-entropy cantor alloys. *Prog. Mater. Sci.* **120**, 100754 (2021).

[28] Zhang, Y. High-Entropy Materials: A Brief Introduction. Springer, 2019.

[29] Kotsonis, G. N. et al. High-entropy oxides: harnessing crystalline disorder for emergent functionality. *J. Am. Ceram. Soc.* **106**, 5587–5611 (2023).

[30] van de Walle, A. & Ceder, G. The effect of lattice vibrations on substitutional alloy thermodynamics. *Rev. Mod. Phys.* **74**, 11–45 (2002).

[31] Sklar, N. & Post, B. Crystal structure of lithium aluminum hydride. *Inorg. Chem.* **6**, 669–671 (1967).

[32] Fallas, J. C. et al. Raman spectroscopy measurements of the pressure and temperature behavior of lialh4. *J. Phys. Chem. C.* **114**, 11991–11997 (2010).

[33] Amsler, M. et al. Novel structural motifs in low energy phases of LiAlH_4_. *Phys. Rev. Lett.* **108**, 205505 (2012). 10.1103/PhysRevLett.108.205505 23003156

[34] Sun, J., Ruzsinszky, A. & Perdew, J. Strongly constrained and appropriately normed semilocal density functional. *Phys. Rev. Lett*. **115**, 036402 (2015). 10.1103/PhysRevLett.115.036402 26230809

[35] Furness, J. W., Kaplan, A. D., Ning, J., Perdew, J. P. & Sun, J. Accurate and numerically efficient R2SCAN meta-generalized gradient approximation. *J. Phys. Chem. Lett.* **11**, 8208–8215 (2020). 10.1021/acs.jpclett.0c02405 32876454

[36] Zhao, X.-G., Dalpian, G. M., Wang, Z. & Zunger, A. Polymorphous nature of cubic halide perovskites. *Phys. Rev. B* **101**, 155137 (2020).

[37] Stillinger, F. H. & Weber, T. A. Hidden structure in liquids. *Phys. Rev. A* **25**, 978–989 (1982).

[38] Stillinger, F. H. Energy Landscapes, Inherent Structures, and Condensed-Matter Phenomena. Princeton University Press, 2016.

[39] Hew, N. L. E., Shang, S.-L. & Liu, Z.-K. Predicting phase transitions in PbTio_3_ using zentropy through quasiharmonic phonon calculations. *Phys. Rev. B* **110**, 184103 (2024).

[40] Rostami, S., Ghasemi, S. A. & Goedecker, S. Novel polymorphs and polytypes of lithium chloride from structure predictions based on charge equilibration via neural network technique. *Phys. Rev. Mater.* **5**, 123603 (2021).

[41] Krummenacher, M. et al. Performing highly efficient minima hopping structure predictions using the atomic simulation environment (ASE). *SoftwareX* **25**, 101632 (2024).

[42] Zhu, L. et al. A fingerprint based metric for measuring similarities of crystalline structures. *J. Chem. Phys.* **144**, 034203 (2016). 10.1063/1.4940026 26801027

[43] Batzner, S. et al. E (3)-equivariant graph neural networks for data-efficient and accurate interatomic potentials. *Nat. Commun.* **13**, 2453 (2022). 10.1038/s41467-022-29939-5 PMC9068614 35508450

[44] Kresse, G. & Hafner, J. Ab initio molecular dynamics for liquid metals. *Phys. Rev. B* **47**, 558–561 (1993). 10.1103/physrevb.47.558 10004490

[45] Kresse, G. & Hafner, J. Norm-conserving and ultrasoft pseudopotentials for first-row and transition elements. *J. Condens. Matter Phys.* **6**, 8245 (1994).

[46] Kresse, G. & Furthmüller, J. Efficiency of ab-initio total energy calculations for metals and semiconductors using a plane-wave basis set. *Comput. Mater. Sci.* **6**, 15–50 (1996). 10.1103/physrevb.54.11169 9984901

[47] Kresse, G. & Furthmüller, J. Efficient iterative schemes for ab initio total-energy calculations using a plane-wave basis set. *Phys. Rev. B* **54**, 11169–11186 (1996). 10.1103/physrevb.54.11169 9984901

[48] Kresse, G. & Joubert, D. From ultrasoft pseudopotentials to the projector augmented-wave method. *Phys. Rev. B* **59**, 1758–1775 (1999).

[49] Pomrehn, G. S., Toberer, E. S., Snyder, G. J. & van de Walle, A. Entropic stabilization and retrograde solubility in Zn_4_sb_3_. *Phys. Rev. B* **83**, 094106 (2011).

[50] Larsen, A. H. et al. The atomic simulation environment—a Python library for working with atoms. *J. Condens. Matter Phys.* **29**, 273002 (2017). 10.1088/1361-648X/aa680e 28323250

[51] Wales, D. Energy Landscapes: Applications to Clusters, Biomolecules and Glasses. Cambridge University Press, 2004.

[52] Calvo, F., Doye, J. & Wales, D. Equilibrium properties of clusters in the harmonic superposition approximation. *Chem. Phys. Lett.* **366**, 176–183 (2002).

[53] Huan, T. D. et al. Low-energy polymeric phases of alanates. *Phys. Rev. Lett.* **110**, 135502 (2013). 10.1103/PhysRevLett.110.135502 23581335

[54] Plimpton, S. Fast parallel algorithms for short-range molecular dynamics. *J. Comput. Phys.* **117**, 1–19 (1995).

[55] Thompson, A. P. et al. Lammps - a flexible simulation tool for particle-based materials modeling at the atomic, meso, and continuum scales. *Comput. Phys. Commun.* **271**, 108171 (2022).

[56] Parrinello, M. & Rahman, A. Polymorphic transitions in single crystals: a new molecular dynamics method. *J. Appl. Phys.* **52**, 7182–7190 (1981).

[57] Behler, J. & Parrinello, M. Generalized neural-network representation of high-dimensional potential-energy surfaces. *Phys. Rev. Lett.* **98**, 146401 (2007). 10.1103/PhysRevLett.98.146401 17501293

[58] Behler, J. Atom-centered symmetry functions for constructing high-dimensional neural network potentials. *J. Chem. Phys*. **134**, 074106 (2011). 10.1063/1.3553717 21341827

[59] Singraber, A. n2p2—the neural network potential package. https://github.com/CompPhysVienna/n2p2 (2018). Version 1.0.0, Vienna, accessed October 12, 2018.

[60] Momma, K. & Izumi, F. Vesta 3 for three-dimensional visualization of crystal, volumetric and morphology data. *J. Appl. Crystallogr.* **44**, 1272–1276 (2011).

