# Supplementary material for: Quantifying entropic stabilization of the structurally tolerant ionic phase of lithium alanate
Source: Commun Phys. 2026 Jun 18;9(1):315. doi: 10.1038/s42005-026-02726-0 (PMC13638467; doi:10.1038/s42005-026-02726-0)
Supplement: Supplementary file 1 [file 42005_2026_2726_MOESM1_ESM.pdf]

Supplementary information to "Quantifying entropic stabilization of the structurally tolerant ionic phase of lithium alanate"

Omer Tayfuroglu, Marco Krummenacher, Jonas A. Funkler, Hannes Huber and Stefan Goedecker

Supplementary Note 1: Results of Machine-Learned Potential Development and Supplementary Figures

For this work, two types of machine-learned potentials (MLPs), namely HDNNPs [1] and NequIP [2], were developed using an iterative process. As shown in Figure S1 both machine learned potentials give energies and forces that are in good agreement with the DFT values. In particular, the important vibrational free energies calculated by VASP [3–7] and NequIP are in very good agreement as can be seen from Fig S2. To be sure that our results are not artifacts of a machine learned potential that is inaccurate in certain regions of configurational space, we have constructed two completely independent potentials and performed the relevant calculations with both of them. As shown in Figure S1, both potentials give accurate forces and energies. The root mean square errors for the NequIP potential are 0.96 meV/atom for the energy and 178 meV/Åfor the forces in the training set and 0.96 meV/atom and 178 meV/Åfor the validation set. For the n2p2 potential the root mean square errors are 0.72 meV and 171 meV/Åfor the training set and 0.25 meV and 177 meV/Åfor the validation set. Some geometry optimizations failed using the n2p2 potential while the optimization always worked using the NequIP potential. We added these non converged structures to the correlation plot which is depicted in Figure S1. Although the n2p2 and the NequIP potential show comparable errors during the training procedure, the additions shows that the n2p2 potential is extrapolating less accurately the forces resulting in severe errors in the forces.

To calculate the vibrational free energies, accurate phonon frequencies are required. Figure S2 shows that there is also good agreement between machine-learning potentials and DFT. In particular, the cross over points where the ionic structure becomes lower in energy than the polymeric structure agrees to within a few Kelvin.

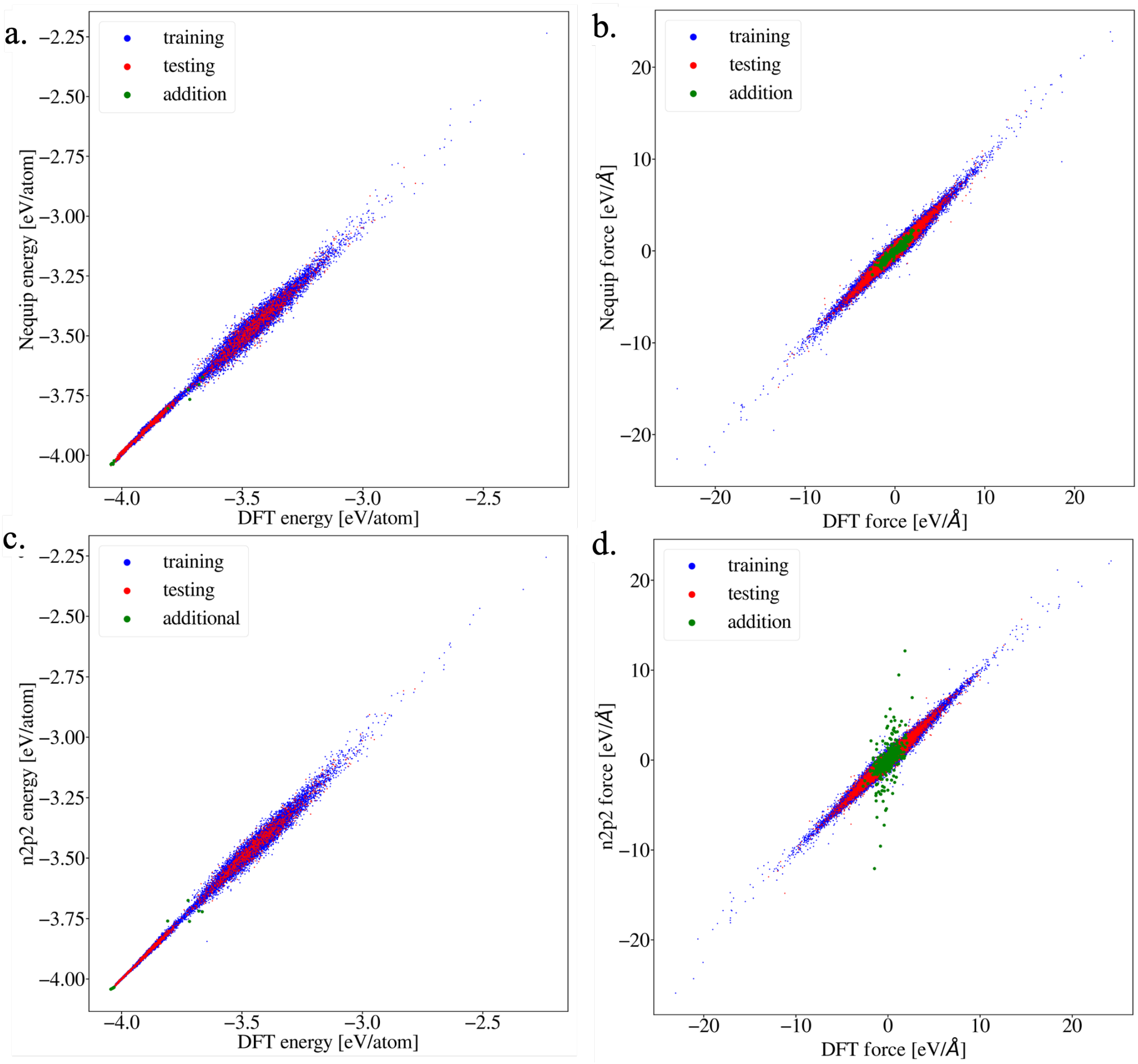

Fig. S1 Accuracy of the machine learned energies and forces: Panel a): NequIP energies vs DFT energies, panel b): NequIP forces vs DFT forces, panel c): n2p2 energies vs DFT energies, panel d): n2p2 forces vs DFT forces. In green the additional dataset is depicted which consists of structures that failed to converge in an n2p2 based geometry optimization.

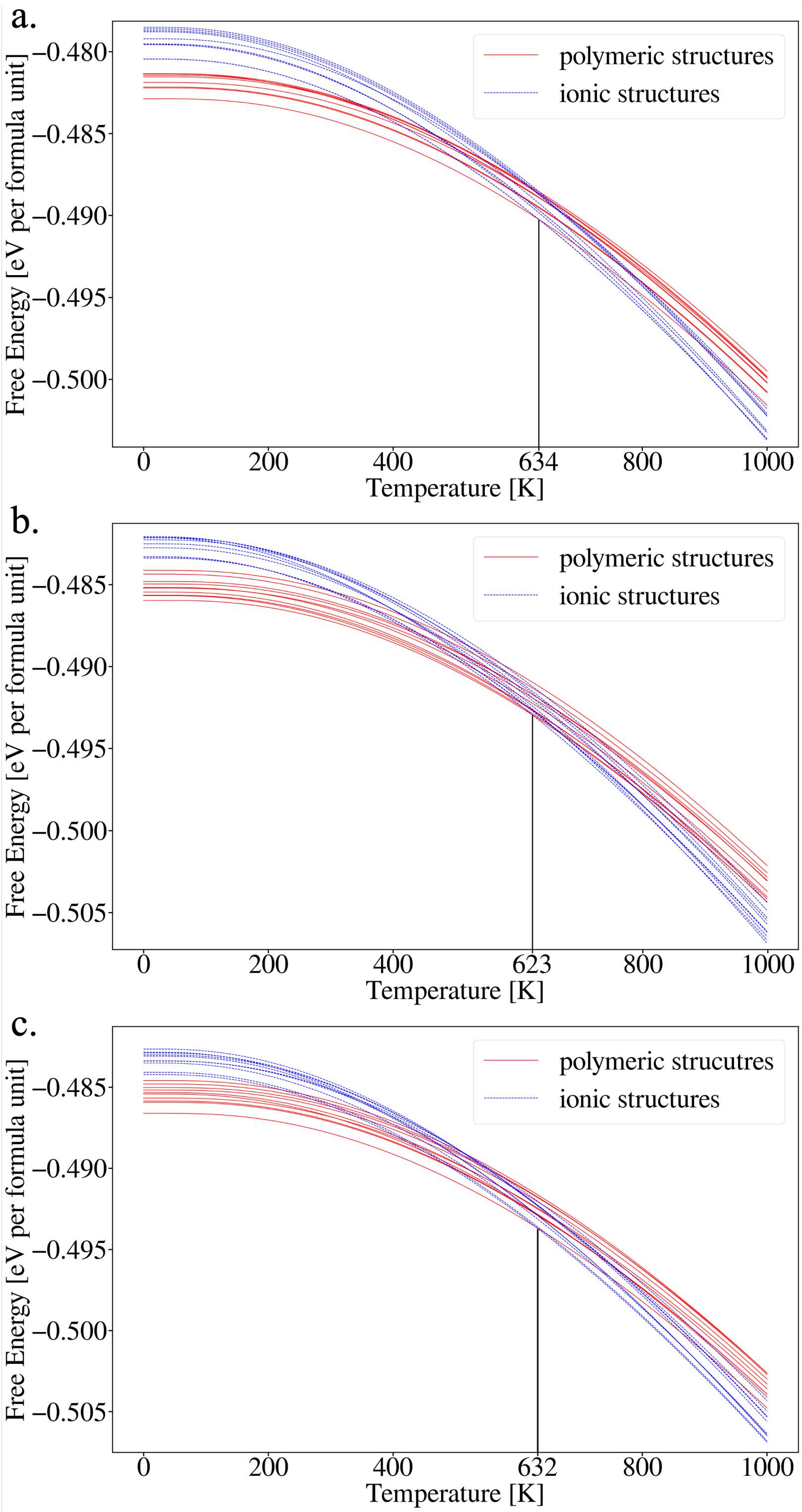

Fig. S2 Free energies within the harmonic approximation of the lowest 10 ionic and polymeric structures: a. n2p2 potential, b. NequIP potential and c. vasp SCAN calculations.

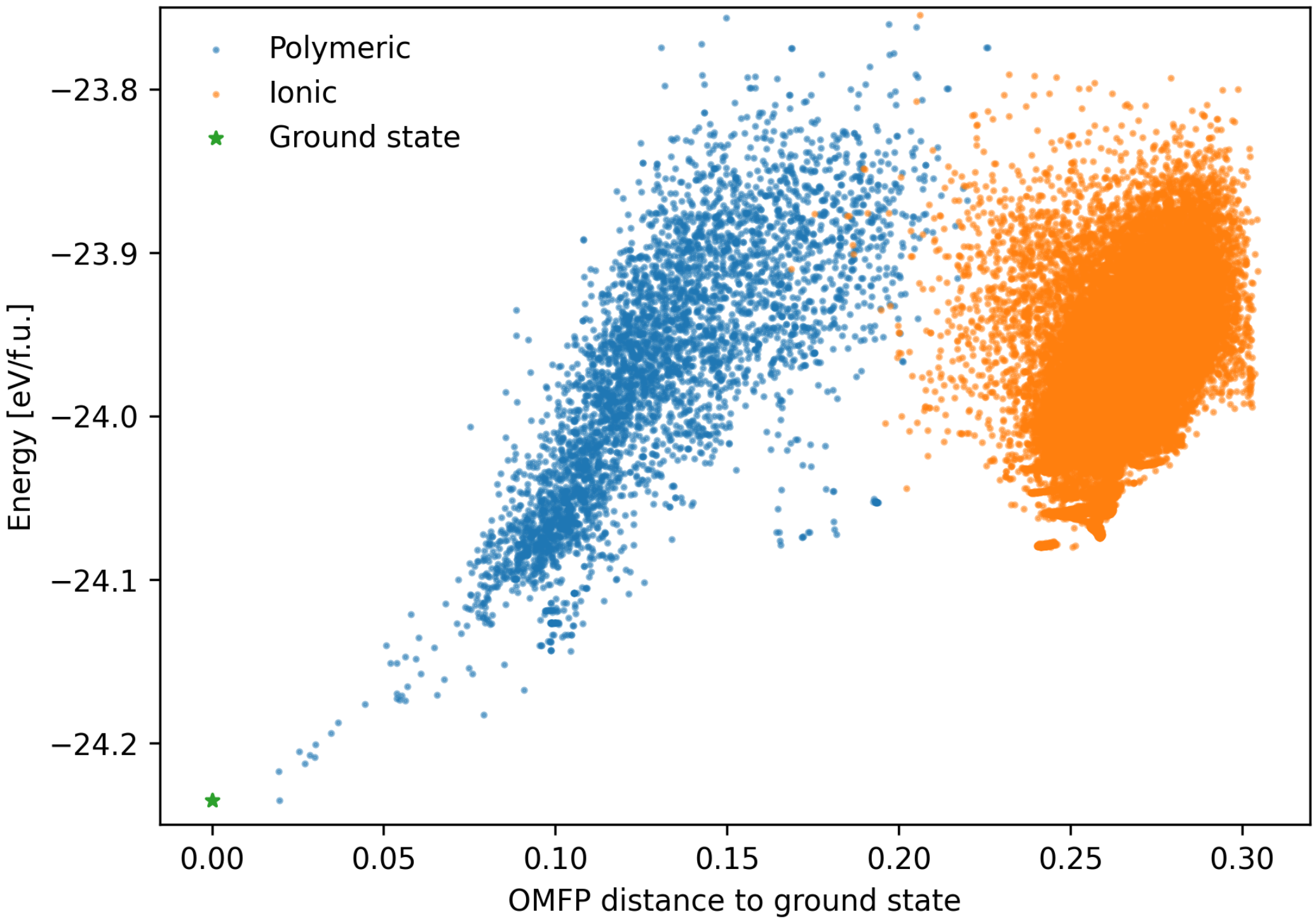

Fig. S3 Overlap matrix fingerprint (OMFP) distances [8] and energy differences with respect to the ground state denoted by a green star for our entire data set. The energy and fingerprint distances correlate well and the polymeric (blue) and ionic (orange) configurations are well separated.

Supplementary Note 2: A simple analytical model

We consider a crystalline system with two phases, A and B. The first phase A has only a non-degenerate ground state at energy zero, shown by the red line in Figure S5. Its defective structures are very high in energy and therefore not taken into account. The second phase B consists of an ensemble of M similar structures that are nearly degenerate in energy and whose energies are clustered in the energy interval $e_0 - 1/2 : e_0 + 1/2$ as shown by the blue density of states in Figure S5. The resulting configurational density of states for the system gives the number of metastable structures per unit energy as a function of the energy. In the following, we will refer to the second phase as structurally tolerant, because it allows for many variations of its basic structural motif that change the energy only weakly. We now calculate the partition functions for both phases. The partition function for phase A is just one because it contains a single structure with zero energy. The partition function Z_B for polymorph B is given by

$$Z_B(\beta) = \int_0^\infty \exp(-\beta\epsilon) D_1(\epsilon) d\epsilon = \frac{2M}{\beta} \exp(-\beta e_0) \sinh(\beta/2) \tag{1}$$

where $\beta = 1/(k_b T)$ and where we used the model configurational density of states

$$D_1(\epsilon) = \begin{cases} M \text{ if } e_0 - 1/2 \leq e_0 + 1/2 \\ 0 \quad \text{else} \end{cases} \tag{2}$$

The Boltzmann probability $p_A(T)$ of finding phase A is then given by the ratio of the partition function of phase A and the sum of the partition functions for A and B:

$$p_A(T) = \frac{1}{1 + Z_B} \tag{3}$$

Of particular interest is the transition temperature where the probabilities of finding polymorphs A and B are equal. This is the case at the inverse temperature β_0 where

$$Z_B(\beta_0) = 1 \tag{4}$$

Inserting the expression from Eq. 1 in the above equation gives

$$\frac{2}{\beta} \exp(-\beta e_0) \sinh(\beta/2) = \frac{1}{M} \tag{5}$$

Since the function $\frac{2}{\beta} \exp(-\beta e_0) \sinh(\beta/2)$, shown in Figure S4 , decreases monotonically from 1 to 0 with increasing β, Eq. 5 has a unique solution β_0 for any positive integer M.

It also follows that β_0 increases with increasing M. This means that one has to go to lower temperatures to observe phase A if the number of structures M in phase B increases. So the probability of finding phase A depends not only on the energy of the polymorphs in this phase but also on the number of defective structures in the competing phase B.

Let us consider now the configurational density of states $D_2(\epsilon)$ of a system that is twice as large as our original system and let us assume that both systems are large and approach the thermodynamic limit. The configurational density of states of the larger system is then related to the configurational density of states of the smaller system by

$$D_2(\epsilon) = \int_{-\infty}^\infty D_1(\epsilon - \epsilon') D_1(\epsilon') d\epsilon' \tag{6}$$

which gives

$$D_2(\epsilon) = \begin{cases} M^2 \left[\epsilon - ((2\epsilon_0 - 1)\right] & \text{if } 2e_0 - 1 \leq \epsilon \leq 2e_0 \\ M^2 \left[(2\epsilon_0 + 1) - \epsilon\right] & \text{if } 2e_0 \leq \epsilon \leq 2e_0 + 1 \\ 0 & \text{else} \end{cases} \tag{7}$$

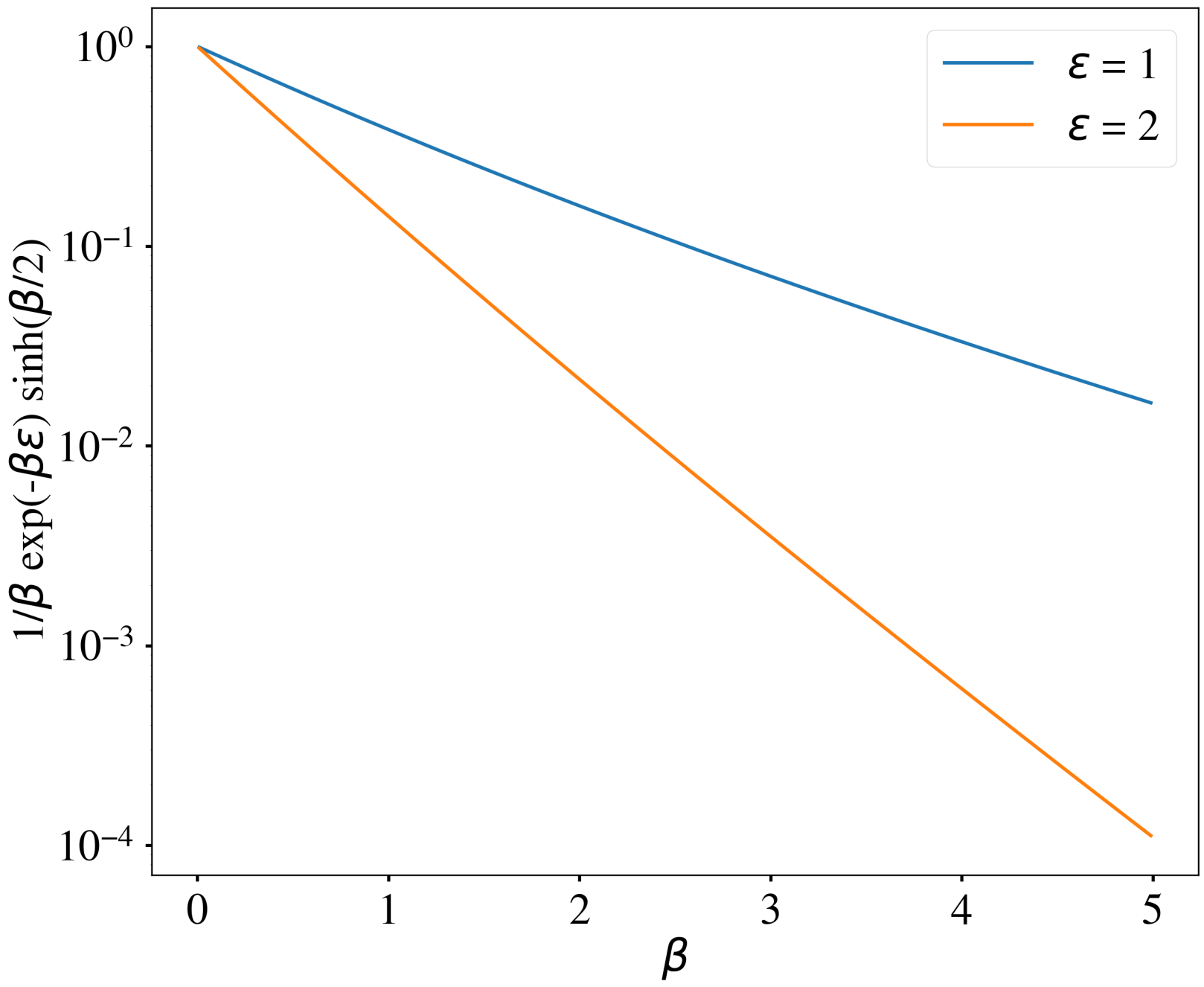

Fig. S4 The function from Eq. 5. Plot of the function on the left hand side of Eq. 4.

The partition function for the larger system, $\tilde{Z}_B$, is then given by

$$\tilde{Z}_B(\beta) = \int_0^\infty \exp(-\beta\epsilon) D_2(\epsilon) d\epsilon = \frac{4M^2}{\beta^2} \exp(-2\beta e_0)(\sinh(\beta))^2 \tag{8}$$

The inverse temperature, where both polymorphs have a probability of 1/2, is then determined by the condition

$$\frac{4}{\beta^2} \exp(-2\beta e_0) \sinh(\beta/2)^2 = \frac{1}{M^2} \tag{9}$$

This equation is identical to Eq. 5 except that both the right and left-hand side are squared. However, since both sides are positive, the solution β_0 is identical for both equations and a temperature where both polymorphs are equally likely to be found now exists and is independent of the system size in the limit of large cells.

Since we want to obtain, in a numerical calculations a transition temperature that is independent of system size, this model system teaches us that we have to investigate a large system that comes sufficiently close to the thermodynamic limit where Eq. 6 is satisfied. If Eq. 6 is satisfied, this also implies that the free energy is varying linearly with respect to the size of the system, i.e. that the free energy of the two times larger system, $F_2(\beta) = \int D_2(\epsilon) \exp(-\beta\epsilon)\, d\epsilon$, is twice the free energy, $F_1(\beta) = \int D_1(\epsilon) \exp(-\beta\epsilon)\, d\epsilon$, of the smaller system. A linear behavior of the free energy is therefore an indicator that the cell is large enough.

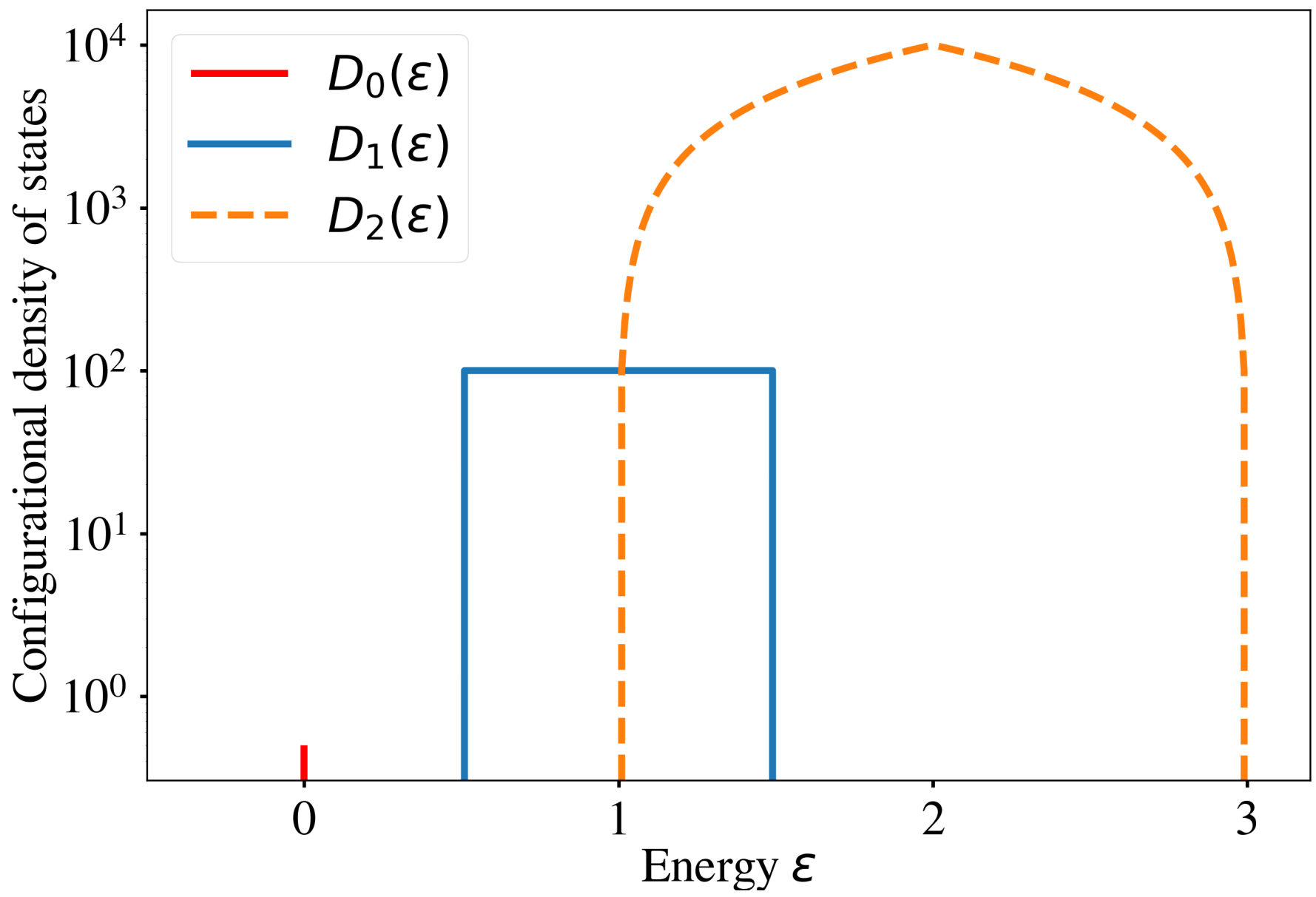

Fig. S5 Configurational density of states for our model system. D_0 is the density of states of polymorph A. Since only one structure exists for this polymorph, the density of states is a delta function as indicated by the red vertical line. D_1 is the density of states for polymorph B in a large supercell and D_2 is the density of states if this cell is doubled in size.

Supplementary References

[1] Behler, J., Parrinello, M.: Generalized neural-network representation of high-dimensional potential-energy surfaces. Phys. Rev. Lett. **98**(14), 146401 (2007) https://doi.org/10.1103/PhysRevLett.98.146401

[2] Batzner, S., Musaelian, A., Sun, L., Geiger, M., Mailoa, J.P., Kornbluth, M., Molinari, N., Smidt, T.E., Kozinsky, B.: E (3)-equivariant graph neural networks for data-efficient and accurate interatomic potentials. Nature communications **13**(1), 2453 (2022) https://doi.org/10.1038/s41467-022-29939-5

[3] Kresse, G., Hafner, J.: Ab initio molecular dynamics for liquid metals. Phys. Rev. B **47**, 558–561 (1993) https://doi.org/10.1103/PhysRevB.47.558

[4] Kresse, G., Hafner, J.: Norm-conserving and ultrasoft pseudopotentials for first-row and transition elements. J. Condens. Matter Phys. **6**(40), 8245 (1994) https://doi.org/10.1088/0953-8984/6/40/015

[5] Kresse, G., Furthmüller, J.: Efficiency of ab-initio total energy calculations for metals and semiconductors using a plane-wave basis set. Comput. Mater. Sci. **6**(1), 15–50 (1996) https://doi.org/10.1016/0927-0256(96)00008-0

[6] Kresse, G., Furthmüller, J.: Efficient iterative schemes for ab initio total-energy calculations using a plane-wave basis set. Phys. Rev. B **54**, 11169–11186 (1996) https://doi.org/10.1103/PhysRevB.54.11169

[7] Kresse, G., Joubert, D.: From ultrasoft pseudopotentials to the projector augmented-wave method. Phys. Rev. B **59**, 1758–1775 (1999) https://doi.org/10.1103/PhysRevB.59.1758

[8] Zhu, L., Amsler, M., Fuhrer, T., Schaefer, B., Faraji, S., Rostami, S., Ghasemi, S.A., Sadeghi, A., Grauzinyte, M., Wolverton, C., *et al.*: A fingerprint based metric for measuring similarities of crystalline structures. J. Chem. Phys. **144**(3), 034203 (2016) https://doi.org/10.1063/1.4940026
